# Deciphering the Mechanism of Melatonin-Induced Enhancement of Photosystem II Function in Moderate Drought-Stressed Oregano Plants

**DOI:** 10.3390/plants13182590

**Published:** 2024-09-16

**Authors:** Julietta Moustaka, Ilektra Sperdouli, Sumrunaz İşgören, Begüm Şaş, Michael Moustakas

**Affiliations:** 1 Department of Botany, Aristotle University of Thessaloniki, 54124 Thessaloniki, Greece; ioumoustaka@gmail.com; 2 Institute of Plant Breeding and Genetic Resources, Hellenic Agricultural Organisation-Demeter (ELGO-Demeter), 57001 Thessaloniki, Greece; esperdouli@elgo.gr; 3 Department of Molecular Biology and Genetics, Istanbul Kültür University, Ataköy 7-8-9-10, 34158 Bakırköy, Turkey; isgorensn@gmail.com; 4 School of Life Sciences, Faculty of Biotechnology, ITMO University, Kronverkskiy Prospekt 49, 197101 Saint-Petersburg, Russia; begum.sas99@gmail.com

**Keywords:** chlorophyll content, non-photochemical quenching, electron transport, quantum yield of PSII photochemistry, singlet-excited oxygen, redox state, excess excitation energy, photoinhibition

## Abstract

Melatonin (MT) is considered as an antistress molecule that plays a constructive role in the acclimation of plants to both biotic and abiotic stress conditions. In the present study, we assessed the impact of 10 and 100 μM MT foliar spray, on chlorophyll content, and photosystem II (PSII) function, under moderate drought stress, on oregano (*Origanum vulgare* L.) plants. Our aim was to elucidate the molecular mechanism of MT action on the photosynthetic electron transport process. Foliar spray with 100 μM MT was more effective in mitigating the negative impact of moderate drought stress on PSII function, compared to 10 μM MT. MT foliar spray significantly improved the reduced efficiency of the oxygen-evolving complex (OEC), and PSII photoinhibition (F*v*/F*m*), which were caused by drought stress. Under moderate drought stress, foliar spray with 100 μM MT, compared with the water sprayed (WA) leaves, increased the non-photochemical quenching (NPQ) by 31%, at the growth irradiance (GI, 205 μmol photons m^−2^ s^−1^), and by 13% at a high irradiance (HI, 1000 μmol photons m^−2^ s^−1^). However, the lower NPQ increase at HI was demonstrated to be more effective in decreasing the singlet-excited oxygen (^1^O_2_) production at HI (−38%), in drought-stressed oregano plants sprayed with 100 μM MT, than the corresponding decrease in ^1^O_2_ production at the GI (−20%), both compared with the respective WA-sprayed leaves under moderate drought. The reduced ^1^O_2_ production resulted in a significant increase in the quantum yield of PSII photochemistry (Φ*_PSII_*), and the electron transport rate (ETR), in moderate drought-stressed plants sprayed with 100 μM MT, compared with WA-sprayed plants, but only at the HI (+27%). Our results suggest that the enhancement of PSII functionality, with 100 μM MT under moderate drought stress, was initiated by the NPQ mechanism, which decreased the ^1^O_2_ production and increased the fraction of open PSII reaction centers (q*p*), resulting in an increased ETR.

## 1. Introduction

Despite constant improvements, increase in crop yields is currently not sufficient to avoid food shortages by 2050 [1,2]. Worldwide food production is estimated to be reduced by 11–25% by the end of the century [3]. Drought stress, when compared to other stresses, is the major problem associated with climate change [4,5,6,7,8], lowering drastically global crop production [9,10], and resulting, e.g., in 21% yield decreases in wheat and in even more, in 40% in maize [11].

Drought causes numerous morphophysiological disorders in plants [12], resulting in reduction in leaf area and plant biomass, in leaf chlorosis and wilting, and in abortion of leaves and flowers, with a subsequent decline in productivity [13,14,15]. The closure of stomata under drought, which reduces transpiration in order to avoid dehydration, decreases CO_2_ disposal and, as a result, the use of electrons for carbon fixation [16,17,18]. Subsequently, the electrons in the light reactions divert to oxygen, thus creating the superoxide anion radical (O_2_^•−^) [18,19,20]. Moreover, the transfer of the excess absorbed light energy from the triplet-excited state of chlorophylls (^3^Chl*) to molecular oxygen (O_2_) creates the singlet oxygen (^1^O_2_), which can further produce O_2_^•−^ and hydrogen peroxide (H_2_O_2_) [21,22,23].

The reactive oxygen species (ROS), which are produced in the light reactions (^1^O_2_, H_2_O_2_, and O_2_**^•^**^−^), are formed persistently, but are kept in homeostasis by enzymatic and non-enzymatic antioxidants under non-stress conditions [24,25,26,27]. However, under drought stress, the homeostasis between the creation and elimination of ROS breakdowns [7], and the generation of ROS rises remarkably [18], initiating oxidative stress that triggers membrane damages, protein degradation and enzyme inactivation, causing damage to cellular components [28,29]. Thus, in drought stress conditions, the excess absorbed light energy that cannot be used in the Calvin–Benson–Bassham cycle results in the disruption of ROS homeostasis [20,21] and, even with the presence of photoprotective mechanisms, the generation of ROS that rises results in photooxidative damage [30,31,32,33]. ROS-provoked oxidative damage under drought stress is one of the key factors that limit plant growth [34,35,36]. Osmolyte accumulation under drought stress conditions confers plant tolerance if it is supplemented by a controlled drought-induced ROS generation [37,38,39]. A non-photochemical quenching (NPQ) mechanism protects the chloroplasts from the damaging effects of ROS by dissipating the excess light energy as heat [40,41,42].

Drought stress impairs cell division, elongation and differentiation, and the osmotic adjustment, triggering loss of turgor [43,44]. It impairs photosynthetic efficiency and plant growth, disturbing light energy partitioning, and finally decreasing plant productivity [43,44]. The primary process in increasing plant productivity is photosynthesis; thus, enhancing photosynthetic efficiency can improve crop yield [45,46]. The use of bio-stimulants against drought stress is the newly adopted strategy to improve photosynthetic efficiency in enhancing crop production, and to overcome threatening agricultural challenges [47,48].

Over the last decade, the agricultural industry has used bio-stimulants as a key approach to stimulate plant growth and enhance crop production under both non-stress and stress conditions [49,50,51,52,53]. The increasing demand for ecological agricultural solutions has positioned bio-stimulants as valuable tools for reinventing farming and sustaining resilience to environmental stressors [54,55]. Applying bio-stimulants mitigates the harmful effects of abiotic stresses, enhancing growth performance and plant tolerance [56]. Bio-stimulants reduce the negative effects of drought stress by lessening damage from oxidative stress [15] and offer a promising and ecological approach to addressing environmental concerns and promoting sustainable agriculture [52]. However, the precise mechanisms by which plant bio-stimulants enhance nutrient uptake and utilization, improve yield, increase photosynthetic function, and confer resistance to both biotic and abiotic stresses have not yet been fully elucidated [50,51,53]. To implement successfully bio-stimulants in the field, an understanding of their action mechanism in coping with diverse abiotic stresses is needed [52].

Melatonin (MT) is a versatile molecule that functions as a natural antioxidant, boosting plants’ tolerance to environmental stresses, and considered as a promising bio-stimulant for agricultural use [57]. MT is considered as a new plant hormone that plays key roles in a broad range of physiological processes, like photosynthesis, leaf senescence, osmoregulation, stomatal closure, circadian cycle, secondary metabolism, germination, shoot and root growth, flowering and fruit setting, and as an anti-stressor [58,59,60,61,62]. As an anti-stress molecule, it plays a constructive role in numerous biotic and abiotic stresses, like drought, salinity, heavy metals, high and low temperatures, plant–pathogen interactions, fungal diseases, and UV radiation [63,64,65,66].

MT can penetrate cell membranes, raising endogenous MT levels [67,68], and acting as an antioxidant under stress conditions [69]. By improving antioxidant capacity and increasing the xanthophyll pool size, MT enhances photosynthetic function and photosynthetic enzyme activities under salt stress [65]. Exogenous MT, under chilling stress, enhanced NPQ, i.e., the photoprotective heat dissipation of excess excitation energy, mitigating photoinhibition [70]. In the medicinal plant *Mentha spicate*, under non-stress conditions, MT stimulated PSII functionality under low light [62]. MT enhanced tolerance to high light in *Arabidopsis thaliana* through improving the anti-oxidative system and photosynthesis [71]. In tomato seedlings MT improved drought tolerance by enhancing soluble sugar accumulation [72]. Soluble sugars, under drought and salt stress, act as osmo-protectants, reducing ROS production and maintaining the cellular redox state [18,39]. MT alleviated abscisic acid deficiency and improved the antioxidant system in rice under salt stress [73]. In maize seeds under salt stress, MT enhanced the antioxidant ability of the seeds, reducing salt-induced damage, and thus promoting seed germination [74]. Under salt stress, exogenous MT led to an increase in endogenous levels, activating the antioxidant system and playing an essential role as ROS scavenger [75]. In cotton, MT, by regulating the expression of photosynthetic genes and the antioxidant system, enhanced cold tolerance [76].

It has been shown that MT preserves chlorophyll molecules and the photosynthetic function [77], regulates the expression of photosynthetic genes [78], and interacts with Ca^2+^, nitric oxide (NO), and ROS, to regulate the redox network [78,79,80,81]. ROS signaling and MT have been shown to be interconnected [65]. Melatonin is related also to the plant hormones, e.g., ethylene (ETH), indole acetic acid (IAA), cyto-kinins (CTK), gibberellins (GAs), abscisic acid (ABA), brassino-steroids (BR), jasmonic acid (JA), strigolactone (SL), and salicylic acid (SA) [82,83].

*Origanum vulgare* L. is a perennial polymorphic species native to the Mediterranean basin, but occurring almost all over Europe and West and Central Asia [84]. It is used both as a medicinal and culinary herb, especially in the Spanish, Mexican, French, Turkish, Italian, and Greek cuisines [62]. In this research work, we tested whether MT foliar spray can enhance PSII function of *Origanum vulgare* L. plants under moderate drought stress and tried to disentangle the underlying mechanisms of MT action on the electron transport process under moderate drought stress conditions.

## 2. Results

### 2.1. Volumetric Soil Water Content and Leaf Water Content

Withholding the irrigation for 6 days resulted in moderate drought-stressed plants, having a soil water content of 50 ± 2% of that of the well-watered plants. At the same time, the leaf water content in drought-stressed water sprayed plants was 87.6 ± 0.4^b^%; in drought-stressed plants sprayed with 10 μM MT, this was 88.1 ± 0.4^b^%, and in drought-stressed plants sprayed with 100 μM MT, t his was 89.6 ± 0.3^a^% (different lower-case letters show significant differences at *p* < 0.05).

### 2.2. Impact of Melatonin on Chlorophyll Content of Drought-Stressed Oregano Leaves

**Figure 1:**
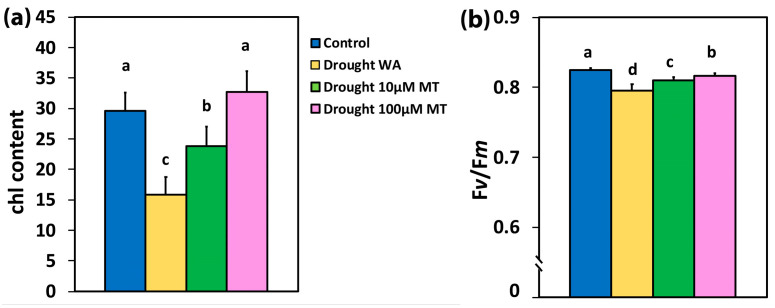
The chlorophyll content, expressed in relative units (**a**), and the maximum efficiency of PSII photochemistry (F*v/*F*m*), in dark adapted leaves (**b**) of control oregano plants, of drought-stressed oregano plants sprayed with water (WA), of drought-stressed oregano plants sprayed with 10 μM MT, and of drought-stressed oregano plants sprayed with 100 μM MT. Error bars are standard deviations (SDs). Significant difference at *p* < 0.05 is shown by different lower-case letters.

A decrease in volumetric soil water content of about 50%, resulted in a significant decrease (47%) in the chlorophyll content of the drought-stressed water sprayed plants (WA) (Figure 1a). In drought-stressed oregano plants sprayed with 10 μM MT (10 μM MT), the chlorophyll content decreased to 80% of that of the well-watered plants (CK)while, in drought-stressed oregano plants sprayed with 100 μM MT (100 μM MT), the chlorophyll content remained at the level of the well-watered plants (CK) (Figure 1a).

### 2.3. Impact of Melatonin on the Maximum Efficiency of Photosystem II Photochemistry and the Efficiency of the Oxygen-Evolving Complex

**Figure 2:**
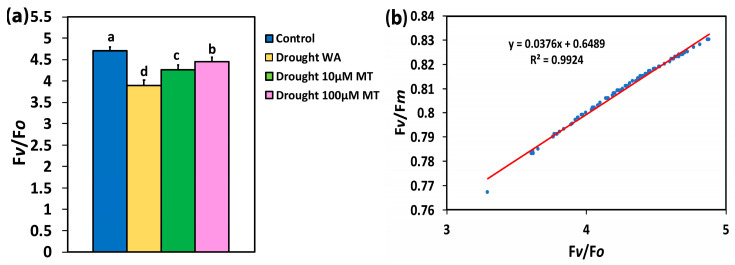
The efficiency of the oxygen-evolving complex (F*v/*F*o*) in dark adapted leaves (**a**), of control oregano plants, of drought-stressed oregano plants sprayed with water (WA), of drought-stressed oregano plants sprayed with 10 μM MT, and of drought-stressed oregano plants sprayed with 100 μM MT. Error bars are standard deviations (SDs). Significant difference at *p* < 0.05 is shown by different lower-case letters. In (**b**), the relationship between the F*v/*F*m*, and the F*v/*F*o* is depicted based on the data from Figure 1b and Figure 2a. A positive significant correlation exists (R^2^ = 0.9924, *p* < 0.001).

In WA plants, the maximum efficiency of PSII photochemistry (*Fv/Fm*) decreased compared to CK plants by 4%, while in 10 μM MT-treated plants this decreased by 2%, and in 100 μM MT plants by 1% (Figure 1b). The efficiency of the oxygen-evolving complex (OEC) in WA plants decreased compared to CK by 17%, while in 10 μM MT this decreased by 10% and in 100 μM MT by 6% (Figure 2a).

The response patterns of F*v/*F*m* and of F*v/*F*o* were similar and, as documented by regression analysis, they showed a positive significant correlation (R^2^ = 0.9924, *p* < 0.001) (Figure 2b).

### 2.4. Impact of Melatonin on Light Energy Use Efficiency of Photosystem II

The absorbed light energy by PSII antenna is either distributed to photochemistry (Φ*_PSII_*), or dissipated as heat (Φ*_NPQ_*), or is misplaced in a nonregulatory way (Φ*_NO_*) [85]. All have a sum equal to 1 [85].

**Figure 3:**
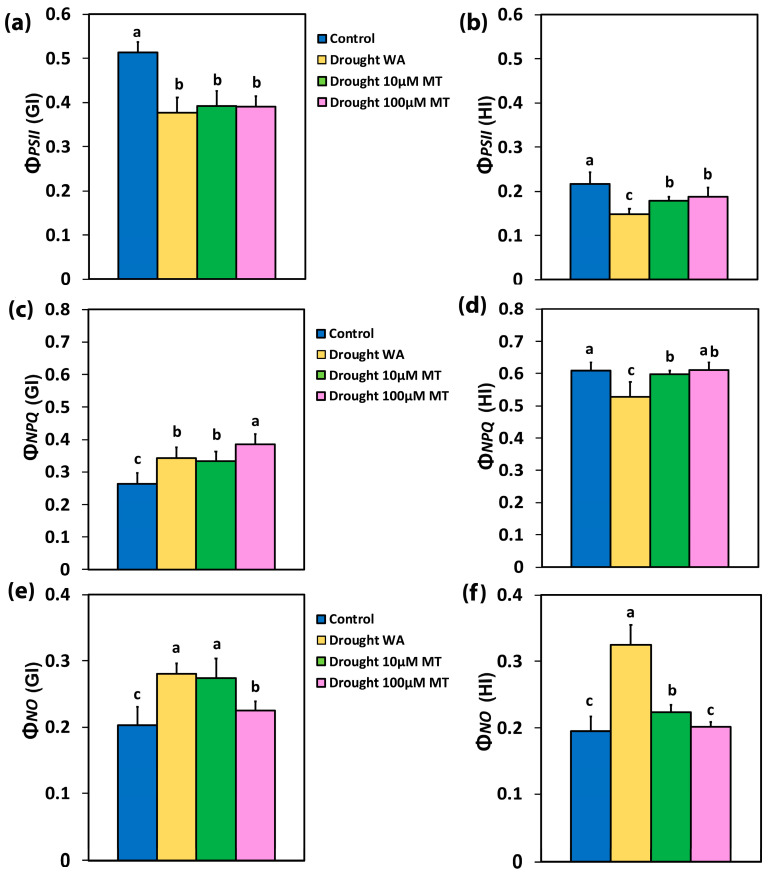
Distribution of the absorbed light energy at PSII. The effective quantum yield of the PSII photochemistry (Φ*_PSII_*) at the growth irradiance (GI) (**a**), and at a high irradiance (HI) (**b**), the quantum yield of regulated non-photochemical energy loss in PSII (Φ*_NPQ_*) at the GI (**c**), and at a HI (**d**), and the quantum yield of non-regulated energy loss in PSII (Φ*_NO_*) at the GI (**e**), and at a HI (**f**); of control oregano plants, of drought-stressed oregano plants sprayed with water (WA), of drought-stressed oregano plants sprayed with 10 μM MT, and of drought-stressed oregano plants sprayed with 100 μM MT. Error bars are standard deviations (SDs). Significant difference at *p* < 0.05 is shown by different lower-case letters.

At the growth irradiance of 205 μmol photons m^−2^ s^−1^ (GI), the quantum yield for the photochemistry (Φ*_PSII_*) of WA oregano plants decreased compared to CK plants by 27%, while the MT-treated plants at both concentrations showed a decrease of 24% (Figure 3a). Similarly, at the high irradiance of 1000 μmol photons m^−2^ s^−^ (HI), Φ*_PSII_* in WA plants decreased compared to CK plants by 32% while, in 10 μM MT and 100 μM MT, there was a lower decrease compared to CK plants, by 18% and 13%, respectively (Figure 3b).

The regulated non-photochemical energy loss in PSII (Φ*_NPQ_*) of WA oregano plants increased (31%) at the GI (Figure 3c), but at HI Φ*_NPQ_* decreased (13%) (Figure 3d), compared to CK plants. In 10 μM MT-treated oregano plants, Φ*_NPQ_* increased (by 27%) at the GI (Figure 3c), but at HI Φ*_NPQ_* decreased (by 2%) (Figure 3d), compared to CK plants. At the GI in 100 μM MT-treated oregano plants, Φ*_NPQ_* increased (by 46%) (Figure 3c), compared to CK plants, but at HI Φ*_NPQ_* remained at the same level as CK plants (Figure 3d).

In WA oregano plants, and in 10 μM MT plants, the yield of non-regulated energy loss in PSII (Φ*_NO_*) increased at the GI by 38%, and 35%, respectively (Figure 3e), while at the HI Φ*_NO_* increased by 66% and 15%, respectively (Figure 3f), compared to CK plants. In 100 μM MT plants, Φ*_NO_* increased (by 11%) at the GI (Figure 3e), compared to CK plants, while at the HI Φ*_NO_* remained at the same level as CK plants (Figure 3e).

### 2.5. Impact of Melatonin on the Photoprotective Heat Dissipation, the Fraction of Open PSII Reaction Centers and the Electron Transport Rate

**Figure 4:**
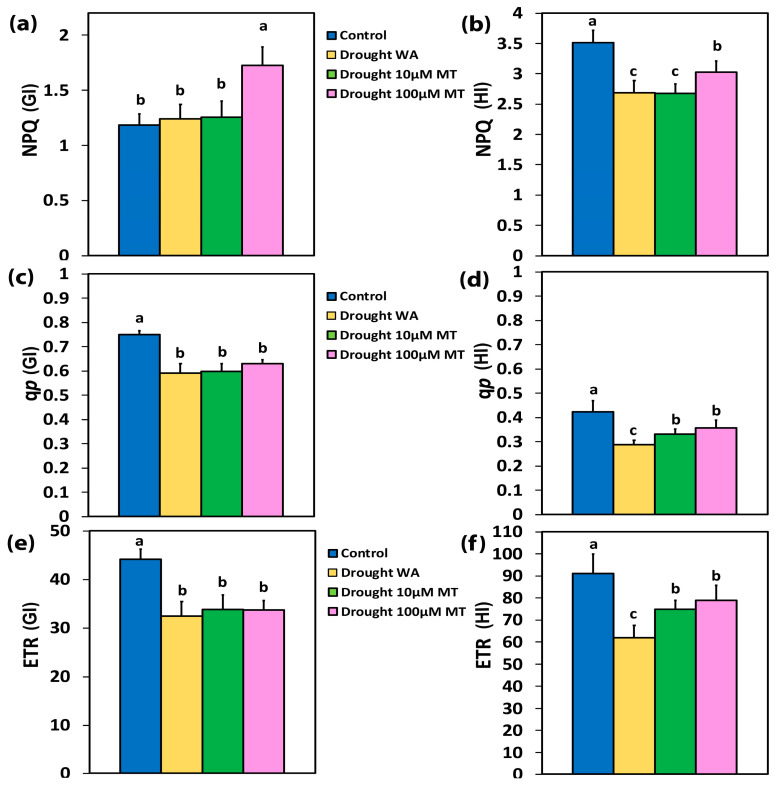
Non-photochemical quenching (NPQ), at the growth irradiance (GI) (**a**), and at high irradiance (HI) (**b**), the fraction of open PSII rection centers (RCs) (q*p*), at the GI (**c**), and at a HI (**d**), and the electron transport rate (ETR) at the GI (**e**), and at a HI (**f**), of control oregano plants, of drought-stressed oregano plants sprayed with water (WA), of drought-stressed oregano plants sprayed with 10 μM MT, and of drought-stressed oregano plants sprayed with 100 μM MT. Error bars are standard deviations (SDs). Significant difference at *p* < 0.05 is shown by different lower-case letters.

The non-photochemical quenching (NPQ), at the GI, in WA plants, and in 10 μM MT plants, remained at the same level as CK plants (Figure 4a). In 100 μM MT-treated plants, NPQ increased (by 46%) at the GI (Figure 4a), compared to CK plants, but at HI NPQ de-creased (by 14%) (Figure 4b). At the HI, NPQ in WA oregano plants, and in 10 μM MT plants, decreased in both by 24%, compared to CK plants (Figure 4b).

The percentage of open PSII rection centers (RCs) (q*p*), which indicates also the redox state of quinone A (Q_A_), in WA plants at 10 μM MT and 100 μM MT, decreased by 21%, 20%, and 16%, respectively at the GI, compared to CK plants (Figure 4c). At the HI, in WA, for 10 μM MT, and 100 μM MT-treated plants, the redox state of Q_A_ decreased by 32%, 22%, and 16%, respectively, compared to CK plants (Figure 4d).

The electron transport rate (ETR), at the GI, in WA plants, in 10 μM MT and 100 μM MT plants, decreased by 27%, 24%, and 24%, respectively, compared to CK plants (Figure 4e). At the HI, ETR in WA plants decreased by 32%, compared to CK plants while, in 10 μM MT, and 100 μM MT-treated plants, ETR decreased by 18%, and 13%, respectively, compared to CK plants (Figure 4f).

### 2.6. Impact of Melatonin on the Efficiency of the Open PSII Reaction Centers and the Excess Excitation Energy at PSII

**Figure 5:**
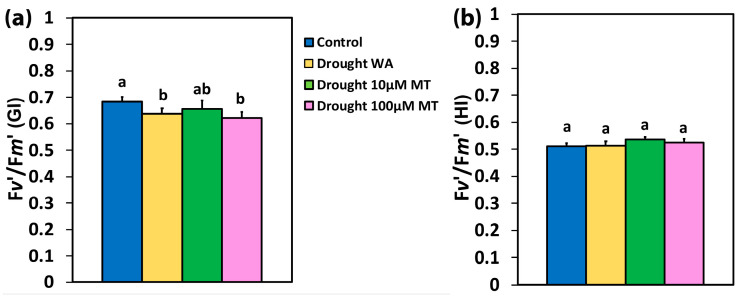
The efficiency of the open PSII RCs (F*v’*/F*m’*), at the growth irradiance (GI) (**a**), and at a high irradiance (HI) (**b**), of control oregano plants, of drought-stressed oregano plants sprayed with water (WA), of drought-stressed oregano plants sprayed with 10 μM MT, and of drought-stressed oregano plants sprayed with 100 μM MT. Error bars are standard deviations (SDs). Significant difference at *p* < 0.05 is shown by different lower-case letters.

The efficiency of the open PSII RCs (F*v’*/F*m’*), at the GI, in WA oregano plants and in 100 μM MT plants, decreased compared to CK plants, but did not change in 10 μM MT plants (Figure 5a). At the HI, F*v’*/F*m’* did not change with any treatment compared to CK plants (Figure 5b).

**Figure 6:**
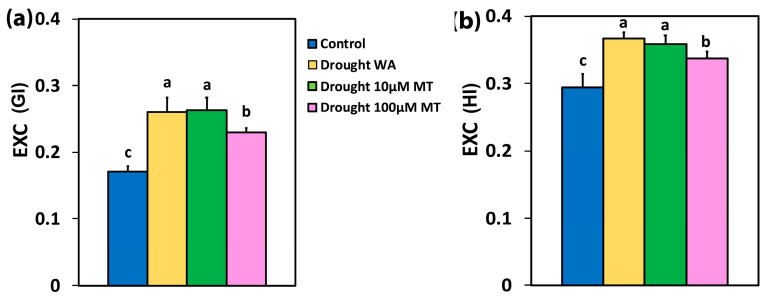
The excess excitation energy at PSII (EXC), at the growth irradiance (GI) (**a**), and at a high irradiance (HI) (**b**), of control oregano plants, of drought-stressed oregano plants sprayed with water (WA), of drought-stressed oregano plants sprayed with 10 μM MT, and of drought-stressed oregano plants sprayed with 100 μM MT. Error bars are standard deviations (SDs). Significant difference at *p* < 0.05 is shown by different lower-case letters.

In WA oregano plants, in 10 μM MT and 100 μM MT plants, the excess excitation energy at PSII (EXC), increased by 52%, 54%, and 35%, respectively, at the GI (Figure 6a) compared to CK plants while, at the HI, the EXC increased by 25%, 22%, and 15%, respectively, compared to CK plants (Figure 6b).

### 2.7. Impact of Melatonin on PSII Excitation Pressure

At the GI, the excitation pressure at PSII (1 − *qL*), in WA plants, in 10 μM MT and 100 μM MT plants, increased by 27%, 29%, and 19%, respectively, compared to CK plants (Figure S1a) while, at the HI, 1 − *qL*, this increased by 14%, 11%, and 8%, respectively, compared to CK plants (Figure S1b).

### 2.8. Impact of Drought and Melatonin on the Spatiotemporal Heterogeneity of PSII Photochemistry

**Figure 7:**
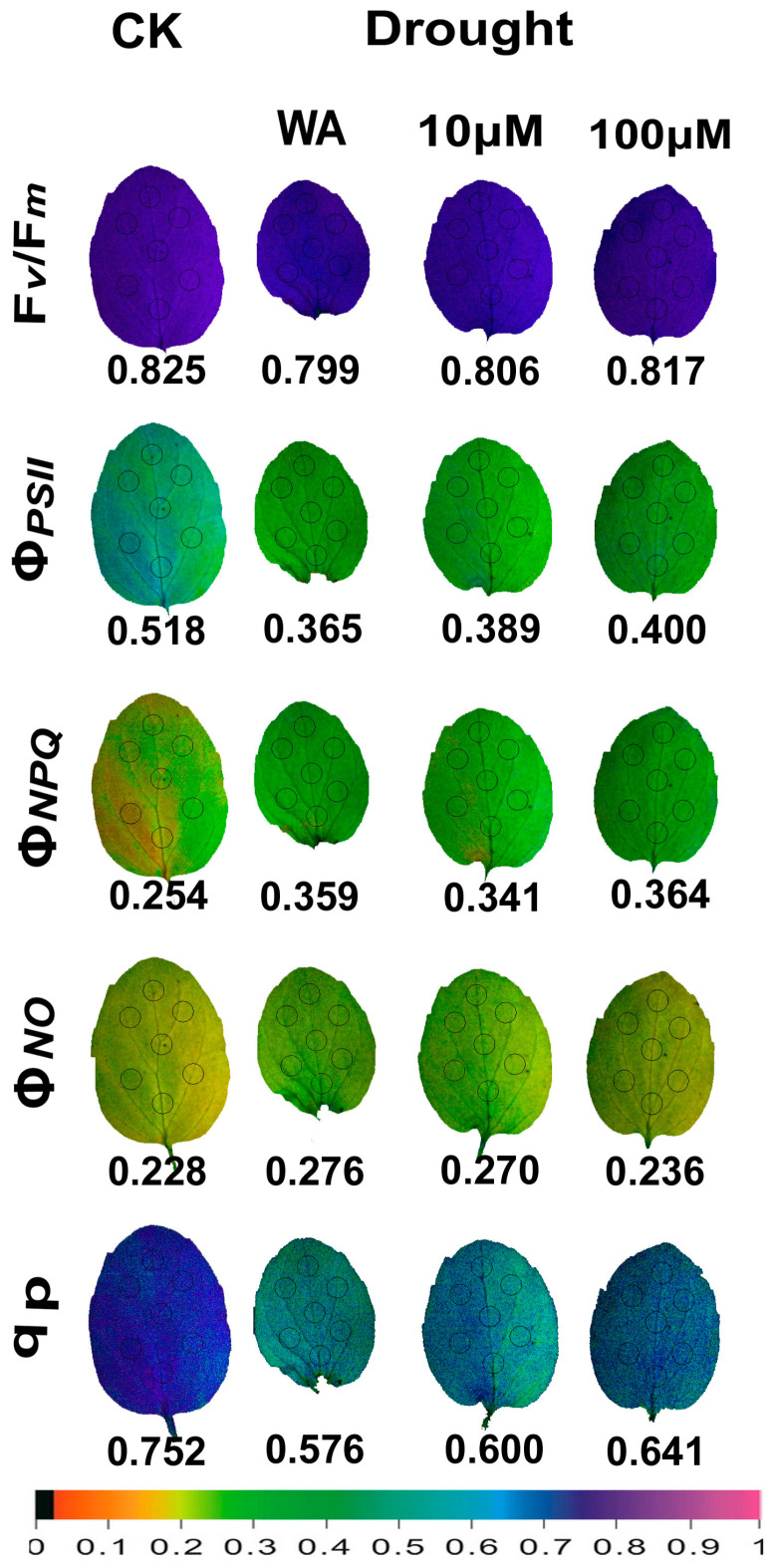
Leaf color-coded pictures of the parameters: F*v/*F*m* captured in dark adapted plants, Φ*_PSII_*, Φ*_NPQ,_* Φ*_NO_*, and q*p*, captured at the growth irradiance (GI), of the control oregano plants (CK), of the drought-stressed oregano plants sprayed with water (WA), of the drought-stressed oregano plants sprayed with 10 μM MT, and of the drought-stressed oregano plants sprayed with 100 μM MT. The seven areas of interest (AOI) that were selected in each leaf are shown by circles. The average whole leaf value for each parameter is shown. At the bottom, the color code indicates the corresponding color values.

Representative color pictures of the whole oregano leaf area that were acquired by chlorophyll fluorescence imaging analysis, for the parameters F*v*/F*m* (captured in dark adapted leaves), and of Φ*_PSII_*, Φ*_NPQ,_* Φ*_NO_*, and q*p* (captured at the growth irradiance, GI), from control oregano plants (CK), drought-stressed oregano plants sprayed with water (WA), drought-stressed oregano plants sprayed with 10 μM MT, and drought-stressed oregano plants sprayed with 100 μM MT are presented in Figure 7. The leaf area of WA plants decreased drastically, while the treatment with 10 μM MT and 100 μM MT mitigated this leaf area decrease (Figure 7). A higher spatial heterogeneity, mainly for the parameters Φ*_PSII_*, Φ*_NPQ,_* and Φ*_NO_*, was noticed in CK oregano plants, compared to all treatments of moderate drought-stressed plants (Figure 7). Moderate drought stress seemed to mask this spatial heterogeneity under all treatments (Figure 7). The negative impact of moderate drought stress, in all parameters, was significantly mitigated mainly by the 100 μM MT treatment (Figure 7).

**Figure 8:**
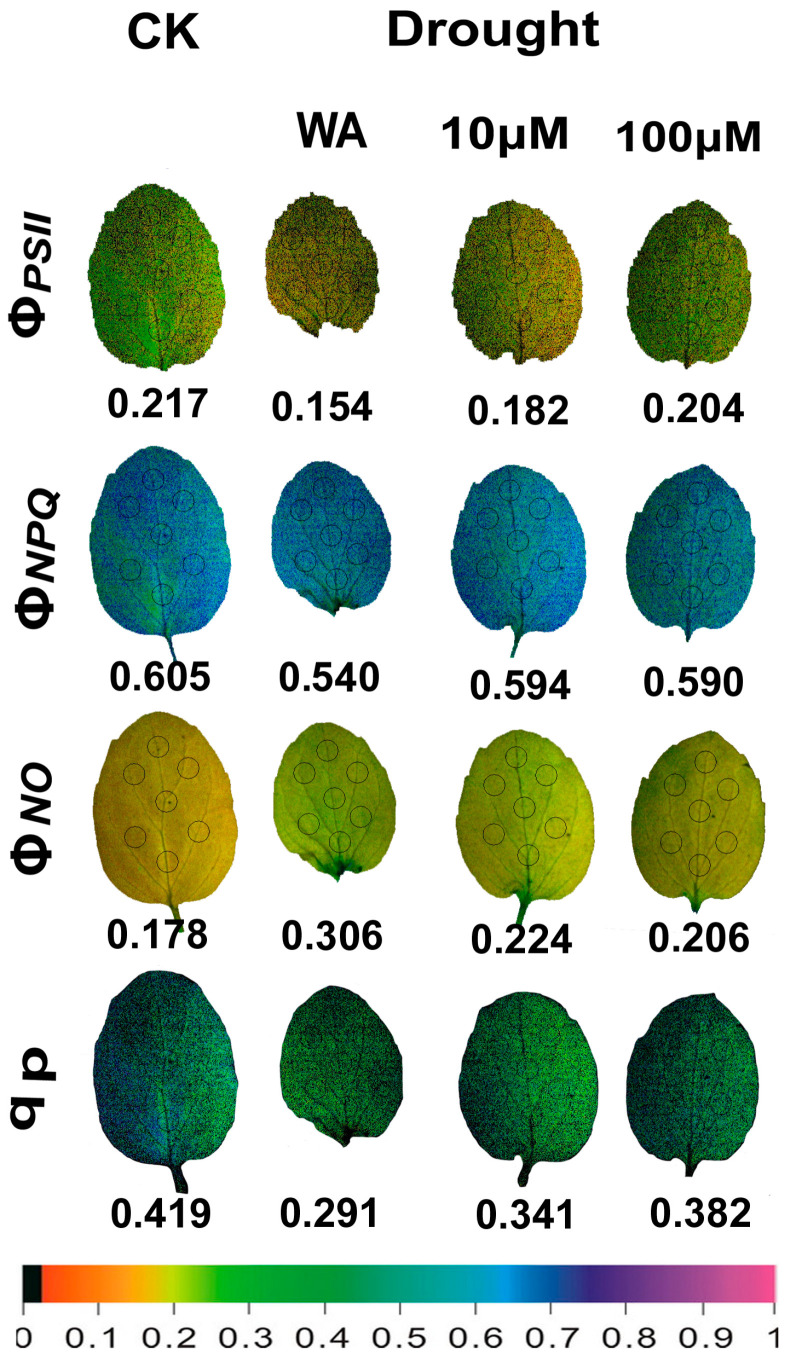
Leaf color-coded pictures of the parameters Φ*_PSII_*, Φ*_NPQ,_* Φ*_NO_*, and q*p*, captured at high irradiance (HI), of the control oregano plants (CK), of the drought-stressed oregano plants sprayed with water (WA), of the drought-stressed oregano plants sprayed with 10 μM MT, and of the drought-stressed oregano plants sprayed with 100 μM MT. The seven areas of interest (AOI) selected in each leaf are shown by circles. The average whole leaf value for each parameter is shown. At the bottom, the color code indicates the corresponding color values.

At high irradiance, (HI), the representative color pictures, which were acquired by chlorophyll fluorescence imaging analysis, of the whole oregano leaf area, for the parameters Φ*_PSII_*, Φ*_NPQ,_* Φ*_NO_*, and q*p*, from CK plants, WA, 10 μM MT and 100 μM MT-treated, are shown in Figure 8. The higher spatial heterogeneity in CK oregano plants, compared to all other treatments (Figure 8), mainly for the parameters Φ*_PSII_*, Φ*_NPQ,_* and q*p*, was still noticed, but was significantly less than that observed at the GI (Figure 7). It seems that the increased dissipation of the absorbed light energy as heat (Φ*_NPQ_*), at the HI compared to the GI, reduced the spatial PSII heterogeneity. The negative impact of moderate drought stress, in all the parameters, was also significantly mitigated at the HI, mainly by the application of 100 μM MT (Figure 8).

## 3. Discussion

Drought stress triggers a decline in CO_2_ availability owing to stomatal closure, leading to the over-reduction of the electron transport process and to increased production of ROS [86,87,88,89,90]. Under moderate drought stress, the percentage of open PSII reaction centers (q*p*) decreased (Figure 4c,d), resulting in a reduced quantum yield of photochemistry (Φ*_PSII_*) (Figure 3a,b), and to increased non-photochemical energy loss in PSII (Φ*_NPQ_*) (Figure 3c) [91,92,93,94,95]. The inability of Φ*_NPQ_* to increase under HI in WA oregano plants (Figure 3d) resulted in an increase in Φ*_NO_* at HI by 66% (Figure 3f), indicating that the photoprotective regulatory mechanisms were insufficient, and that the plants had severe problems in coping with the absorbed light energy [94]. The non-photochemical quenching (NPQ) mechanism assists in dissipating the excess light energy as heat, preventing overexcitation of the chlorophyll molecules, and protecting the photosynthetic apparatus against photodamage under drought stress conditions [96,97,98]. Drought stress impairs photosynthetic efficiency by disturbing the light energy partitioning [7,44].

In our experiments, the decreased Φ*_PSII_* in WA or 10 μM MT plants, compared to CK plants (Figure 3a,b), could not be compensated for by the increased Φ*_NPQ_* (Figure 3c,d) and, as a result, the quantum yields of non-regulated energy loss in PSII (Φ*_NO_*) increased (Figure 3e,f). However, in 100 μM MT plants, the decreased Φ*_PSII_* was balanced by the increase in Φ*_NPQ_*, partially at the GI (Figure 3c), and fully balanced at the HI (Figure 3d), compared to CK plants. As a result, Φ*_NO_* at HI remained at the level of that of the CK plants (Figure 3f). Non-regulated energy dissipated in PSII (Φ*_NO_*) is the result of ^1^O_2_ formation [99,100,101,102]. Thus, the reduced ^1^O_2_ production, which remained at the level of CK plants with 100 μM MT, resulted in a significant increase in Φ*_PSII_* (Figure 3b) and ETR (Figure 4f) by 27%, only at the HI, both parameters being compared with the respective WA ones. Τhis increase in Φ*_PSII_* at the HI, in 100 μM MT, compared with the respective WA (Figure 3b), was due to the 25% increase in the open PSII RCs (Figure 4d), since the excitation capture efficiency of the open PSII RCs (F*v’*/F*m’*) at the HI did not differ (Figure 5b).

Τhe leaf water content of drought-stressed plants sprayed with 100 μM MT was significantly higher from drought-stressed water sprayed plants and drought-stressed plants sprayed with 10 μM MT, suggesting the induction of osmolyte accumulation [72]. In accordance with our results, foliar application of 100 μM MT reversed drought-induced impairment of the leaf water content in tomato seedlings, by increasing the sugar accumulation, particularly sucrose content [72]. Soluble sugars, together with proline, act as osmo-protectants in subsequent periods of drought stress [39,72]. Exogenous MT application under non-stressed conditions in *Chara australis* [103] and in *Mentha spicata* [68] amplified the number of open PSII RCs, thus improving Φ*_PSII_*. In tall fescue under high-temperature stress, MT also increased Φ*_PSII_* by increasing the fraction of open RCs [104]. Under non-stressed conditions, application of MT in *Camellia sinensis* stimulated photosynthesis and alleviated PSII photoinhibition, displaying a direct antioxidant effect [105]. Drought stress is documented to harm the oxygen-evolving complex (OEC) of PSII [106], decreasing the abundance of OEC proteins [89]. Malfunction of the OEC is related to donor side photoinhibition [107,108,109,110].

In our experiments, application of MT relieved photoinhibition (judged from F*v/*F*m*), which was caused by moderate drought stress (Figure 1b). The reduced F*v/*F*m* from drought stress (Figure 1b) was due to donor-side photoinhibition due to malfunction of the OEC [109,110,111]. The malfunction of the OEC, caused by moderate drought stress (Figure 2a), was judged from the ratio F*v/*F*o* [112,113,114,115,116,117,118]. The higher efficiency of the OEC in MT-sprayed oregano plants, compared to WA-sprayed ones (Figure 2a), matched the respective improved maximum efficiency of PSII photochemistry (F*v/*F*m*) (Figure 1b). Reduced efficiency of the OEC leads to a decline in F*v*/F*m* [119,120], as documented by the positive significant correlation in our regression analysis (Figure 2b). A donor-side photoinhibition is often linked with ROS production [111,121,122]. The observed increased ^1^O_2_ generation in moderate drought-stressed plants sprayed either with WA or with 10 μM MT (Figure 3e,f) was possible due to the donor-side photoinhibition (Figure 1b), owing to the malfunction of the OEC (Figure 2a). Application of 100 μM MT significantly alleviated the decreased efficiency of the OEC and PSII photoinhibition caused by moderate drought stress. In contrast, under non-stress conditions, MT reduced the efficiency of the OEC, causing donor-side photoinhibition [62].

The reduction of the excess excitation energy, which was detected with 100 μM MT, at both GI (Figure 6a), and HI (Figure 6b), compared to WA and 10 μM MT, indicates enhanced efficiency of PSII. Enhancing photosynthesis is a major challenge for plant scientists, particularly given the growing global demand for food [123,124,125]. The key to improving photosynthetic efficiency lies in optimizing the distribution of absorbed light energy [94,126].

A decreased excitation pressure at PSII (1-*qL*), at both GI (Figure S1a) and HI (Figure S1b), was detected with 100 μM MT, compared to WA plants. A decreased excitation pressure has been corelated to increased expression level of photosystem II subunit S (PsbS) [127,128], which is associated with increased levels of NPQ [129]. A higher NPQ level is a characteristic of drought-tolerant cultivars [95]. Foliar spray with 100 μM MT, compared with WA leaves, resulted in increased NPQ by 31% at the GI (Figure 4a), and by 13% at the HI (Figure 4b). The lower NPQ increase at HI, with 100 μM MT, compared with WA leaves, was proved to be more effective in decreasing ^1^O_2_ production at the HI by 38% (Figure 3f), than the corresponding decrease in ^1^O_2_ production at the GI by 20% (Figure 3e), both compared with the respective WA leaves.

Whole leaf evaluation of PSII functionality by chlorophyll fluorescence imaging methodology revealed a higher spatial heterogeneity in control oregano plants at the GI, compared to all treatments of drought-stressed plants (Figure 7). However, at HI (Figure 8), this spatial heterogeneity was significantly lower than that observed at the GI (Figure 7). It seems that the increased dissipation of the absorbed light energy as heat (Φ*_NPQ_*), at the HI compared to the GI, reduced the spatial heterogeneity. However, in *Arabidopsis thaliana,* the observed order of the higher spatial PSII heterogeneity was first at mild drought stress, next at moderate drought stress, and with least heterogeneity for control plants [130]. The spatial PSII heterogeneity observed in control oregano plants suggests that water potential and stomatal function differ in cells from different regions of the leaf, contributing to spatial differences in photochemical activity and reflecting different zones of the leaf anatomy and mesophyll development [131,132]. Moderate drought stress in oregano plants seemed to mask the differences, resulting in less spatial heterogeneity (Figure 7).

Foliar application of MT in mint plants, under non-stress conditions, improved PSII functionality by triggering the NPQ mechanism that stimulated ROS production, which enhanced the photosynthetic function [62]. In the present experiment, oregano plants that were sprayed with MT before the moderate drought stress show an enhancement of PSII functionality, initiated by the NPQ mechanism, which decreased ^1^O_2_ production and increased the ETR. It seems that, under different environmental growth conditions, MT application triggers differentially the NPQ mechanism, which activates differential ROS regulatory networks of light energy partitioning signaling to improve PSII function.

## 4. Materials and Methods

### 4.1. Plant Material and Cultivation

Six-week-old oregano (*Origanum vulgare* L.) plants obtained from the Garden Center Vaseiliadis were transported to a growth chamber and transplanted into a 1.5 L pot. Throughout the experimental period the oregano plants were grown at a day/night temperature of 21 ± 1/19 ± 1 °C, a relative humidity during day/night of 60 ± 5/70 ± 5%, and a 14-h photoperiod provided by white fluorescent light lamps with a photosynthetic photon flux density (PPFD) of 200 ± 10 μmol photons m^−2^ s^−1^ [133]. Eight to ten plants were measured from each treatment (n = 8–10).

### 4.2. Melatonin Treatments

In the experiments, we used MT (N-acetyl-5-methoxytryptamine) obtained from Sigma-Aldrich (St. Louis, MO, USA). Twenty mg of MT was dissolved in 1 mL ethanol, before being further diluted with ultra-pure water [62,134] to a final concentration of 10 μM or 100 μM MT [62]. Oregano plants used as control were sprayed with distilled water, with an equal amount of ethanol to that in MT-sprayed plants, as before [62].

### 4.3. Drought Stress Treatment

All oregano plants, after being each transplanted to a 1.5 L pot, were irrigated at full soil water capacity, proceeding to measurements that served as controls. After measurement, oregano plants were foliar-sprayed with 15 mL per plant (until full wetting), with either distilled water (dH_2_O), or 10 μM MT, or 100 μM MT. The surface of the soil was isolated by aluminum foil to prevent MT from dropping into the soil. All plants were sprayed during the dark cycle, since MT may be photo-responsive [135]. The aluminum foil was removed after spraying, and irrigation was withheld in all three groups for 6 days, until soil water content was maintained at 50 ± 2% of the well-watered plants. Four to five plants from each group were measured and two independent experiments were performed (*n* = 8–10).

### 4.4. Soil Water Content

The soil moisture sensor (5TE; Decagon Devices, Pullman, WA, USA), jointly with the ProCheck device (Decagon Devices), was used for measuring the volumetric soil water content [130]. The results are presented as percentage of the full soil water capacity of the well-watered oregano plants.

### 4.5. Leaf Water Content

The water content of oregano leaves was determined by the electronic moisture balance (MOC120H, Shimadzu, Tokyo, Japan) with the formula: (FW–DW) ⁄ DW × 100%, where FW is fresh weight and DW is dry weight [136].

### 4.6. Chlorophyll Content

The chlorophyll content in oregano leaves was measured with a portable chlorophyll content meter (Model Cl-01, Hansatech Instruments Ltd., Norfolk, UK), as described in detail [137]. Results were expressed in relative units [137].

### 4.7. Chlorophyll Fluorescence Imaging Analysis

The modulated Imaging-PAM Fluorometer M-Series (Heinz Walz GmbH, Effeltrich, Germany) was used for chlorophyll fluorescence imaging analysis, performed as described in detail previously [99]. Seven areas of interest (AOI) were selected in each leaf to cover the whole leaf area. The actinic light (AL) used for estimating PSII function was 205 μmol photons m^−2^ s^−1^ (corresponding to growth irradiance, GI) and 1000 μmol photons m^−2^ s^−1^ (corresponding to high irradiance, HI). The measured chlorophyll fluorescence parameters (described in Supplementary Table S1) were estimated using the Win software version 2.32 (Heinz Walz GmbH, Effeltrich, Germany). Color-coded images of selected chlorophyll fluorescence parameters for control (well-watered, water-sprayed, CK), moderate drought-stressed, water sprayed (WA), moderate drought-stressed sprayed with 10 μM MT (10 μM MT) and moderate drought-stressed sprayed with 100 μM MT (100 μM MT) are also presented.

### 4.8. Statistical Analysis

Statistical analysis was performed using the IBM SPSS Statistics for Windows version 28. A one-way ANOVA test was performed to evaluate the effect of the treatment on each photosynthetic parameter, followed by Tukey post-hoc test for pairwise comparisons. Significance was set at a *p* < 0.05 level. A linear regression analysis was also performed. Eight to ten plants were used for statistical analysis for each treatment (n = 8–10).

## 5. Conclusions

Foliar spraying of oregano plants with 100 μM MT was documented to be more effective than 10 μM MT, by retaining higher leaf water content and preserving the chlorophyll content under moderate drought stress, thus mitigating the negative impact on PSII function. MT significantly improved the malfunction of the OEC and the resulting PSII photoinhibition caused by moderate drought stress. It is suggested that, under moderate drought stress, MT exerts its action on oregano plants, by triggering the NPQ mechanism to decrease ^1^O_2_ production, mainly at HI. The reduced ^1^O_2_ production resulted in ameliorating PSII photochemistry and, by increasing the percentage of open PSII reaction centers, ETR was increase. It is concluded that MT may reduce the excess excitation energy by reducing ^1^O_2_ formation, and may also enhance the photosynthetic function of moderate drought-stressed oregano plants. Consequently, it can be regarded as a promising agent for improving the ability of crop plants to face drought episodes in combination with the high light conditions of the Mediterranean area, which influence crop production detrimentally. However, since there is a differential impact of MT on the light energy use efficiency at PSII, depending on the light intensity and the plant species, more experiments must be performed with different crop species to establish the extensive use of MT in agriculture, in order to accomplish sustainable crop production to meet the challenge of drought stress due to climate change.

## Data Availability

The data presented in this study are available in this article.

## Supplementary Materials

The following supporting information can be downloaded at: https://www.mdpi.com/article/10.3390/plants13182590/s1, Figure S1. The excitation pressure at PSII (1 − *qL*). Table S1: Definitions of the chlorophyll fluorescence parameters used in the experiments.
