# Supplementary material for: Deciphering the Mechanism of Melatonin-Induced Enhancement of Photosystem II Function in Moderate Drought-Stressed Oregano Plants
Source: Plants (Basel). 2024 Sep 16;13(18):2590. doi: 10.3390/plants13182590 (PMC11434670; doi:10.3390/plants13182590)
Supplement: Supplementary file 1 [file plants-13-02590-s001.zip › plants-3162534-supplementary.pdf]

plants

MDPI

Article

Deciphering the Mechanism of Melatonin-induced Enhancement of Photosystem II Function in Moderate Drought-stressed Oregano Plants

Julietta Moustaka, Ilektra Sperdouli, Sumrunaz İşgören, Begüm Şaş, and Michael Moustakas

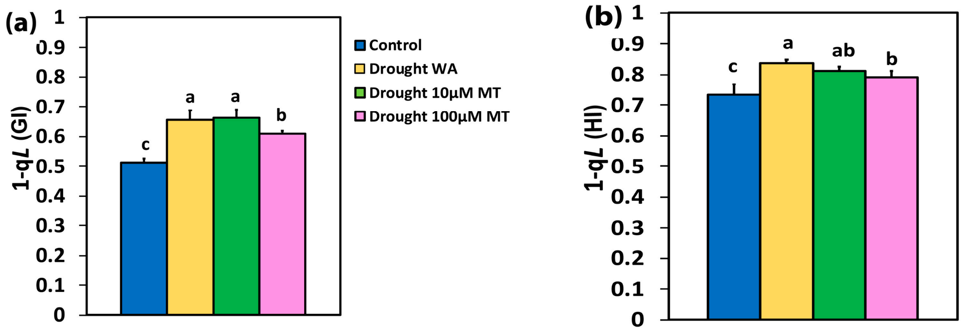

Figure S1. The excitation pressure at PSII (1−*qL*), at the growth irradiance (GI) **(a)**, and at a high irradiance (HI) (**b),** of control oregano plants, of drought stressed oregano plants sprayed with water (WA), of drought stressed oregano plants sprayed with 10 μM MT, and of drought stressed oregano plants sprayed with 100 μM MT. Error bars are standard deviations (SD). Significant difference at $p < 0.05$ is shown by different lower-case letters.

Table S1. Definitions of the chlorophyll fluorescence parameters

Parameter	Definition	Calculation
F*v*/F*m*	Maximum efficiency of PSII photochemistry	(F*m* – F*o*)/F*m*
Φ_{PSII}	Effective quantum yield of PSII photochemistry	(F*m*′ – F*s*)/F*m*′
Φ_{NPQ}	Quantum yield of regulated non-photochemical energy loss in PSII	F*s*/F*m*′ – F*s*/F*m*
Φ_{NO}	Quantum yield of non-regulated energy loss in PSII	F*s*/F*m*
F*v*′/F*m*′	Efficiency of the open PSII reaction centers	(F*m*′ – F*o*′)/F*m*′
F*v*/F*o*	Efficiency of the oxygen evolving complex (OEC) on the donor side of PSII	(F*m* – F*o*)/F*o*
ETR	Electron transport rate	Φ_{PSII} × PAR × c × abs, where PAR is the photosynthetically active radiation, c is 0.5, and abs is the total light absorption of the leaf taken as 0.84
q*p*	Photochemical quenching, representing the redox state of quinone A (Q_A), or in other words the fraction of open PSII reaction centers based on the “puddle” model for the photosynthetic unit	(F*m*′ – F*s*)/(F*m*′ – F*o*′)
NPQ	Non-photochemical quenching reflecting the dissipation of excitation energy as heat	(F*m* – F*m*′)/F*m*′
EXC	Excess excitation energy	$(1 - q_p)$/F*v*′/F*m*′
1-qL	The fraction of closed PSII reaction centres based on the “lake” model for the photosynthetic unit	$1- (q_p \times F_o'/F_s)$
